# Prognostic significance of signal transducer and activator of transcription 5 and 5b expression in Epstein–Barr virus‐positive patients with chronic lymphocytic leukemia

**DOI:** 10.1002/cam4.804

**Published:** 2016-07-01

**Authors:** Panagiotis T. Diamantopoulos, Maria Sofotasiou, Zafiroula Georgoussi, Nefeli Giannakopoulou, Vasiliki Papadopoulou, Athanasios Galanopoulos, Elina Kontandreopoulou, Panagiotis Zervakis, Paschalina Pallaki, Fani Kalala, Marie‐Christine Kyrtsonis, Aglaia Dimitrakopoulou, Theodoros Vassilakopoulos, Maria Angelopoulou, Nikolaos Spanakis, Nora‐Athina Viniou

**Affiliations:** ^1^ First Department of Internal MedicineHematology UnitNational and Kapodistrian University of AthensLaikon General HospitalAthensGreece; ^2^ Laboratory of Cellular Signaling and Molecular PharmacologyInstitute of Biosciences and ApplicationsNational Centre for Scientific Research “Demokritos”AthensGreece; ^3^ Department of Clinical HematologyG. Gennimatas District General HospitalAthensGreece; ^4^ Department of Immunology and HistocompatibilityLaikon General HospitalAthensGreece

**Keywords:** Chronic lymphocytic leukemia, Epstein–Barr virus, latent membrane protein 1, signal transducer and activator of transcription 5

## Abstract

Signal transducer and activator of transcription (STAT) proteins have been intensively studied in hematologic malignancies, and the efficacy of agents against STATs in lymphomas is already under research. We investigated the expression of total STAT5 and STAT5b in peripheral blood samples of patients with chronic lymphocytic leukemia (CLL) in correlation with the presence of Epstein–Barr Virus (EBV) and its major oncoprotein (latent membrane protein 1, LMP1). The EBV load was measured in the peripheral blood by real‐time PCR for the BXLF1 gene and the levels of LMP1 by PCR and ELISA. Western blotting was performed for total STAT5 and STAT5b in protein extracts. STAT5b was only expressed in patients (not in healthy subjects) and STAT5 but particularly STAT5b expression was correlated with the presence of the virus (77.3% vs. 51.2%, *P* = 0.006 for STAT5b) and to the expression of LMP1 (58.3% vs. 21.6%, *P* = 0.011 for STAT5b). Moreover, the expression of STAT5b and the presence of EBV and LMP1 were strongly negatively correlated with the overall survival of the patients (log‐rank test *P* = 0.011, 0.015, 0.006, respectively). Double positive (for EBV and STAT5b) patients had the lowest overall survival (log‐rank test *P* = 0.013). This is the first report of a survival disadvantage of EBV+ patients with CLL, and the first time that STAT5b expression is correlated with survival. The correlation of STAT5 expression with the presence of the virus, along with our survival correlations defines a subgroup of patients with CLL that may benefit from anti‐STAT agents.

## Introduction

The signal transducer and activator of transcription (STAT) proteins are cytosolic transcription factors that, upon activation by extracellular cytokines through receptor‐associated tyrosine kinases, dimerize and migrate to the nucleus to control gene expression. The STAT family comprises of seven members that act to regulate many aspects of survival, growth, and differentiation. STAT5, among them, consists of two isoforms (STAT5A and STAT5B) that are 90% identical at the amino acid level although encoded by two adjacent genes located on 17q11.2 1. Aberrant STAT5 activity has been closely connected to tumorigenesis 2. Inhibition of STAT3 and STAT5 signaling has been shown to arrest the growth of several cancer models 3, and cancer cells are more dependent on STAT activity than normal cells 4. These two features render the STAT family a potential therapeutic target for malignancies, where potent growth inhibition could be coupled with limited toxicity. Therefore, STAT inhibitors have been used in vitro and in animal models to suppress the proliferation of lymphoma cells, and antisense oligonucleotide drugs against STAT proteins have been used in phase 1 and 2 clinical trials against lymphomas with promising results 5.

STAT5 is widely expressed throughout the hematopoietic system and can be activated by a variety of cytokines and growth factors 6, 7, 8. Activation of the JAK‐STAT pathway is a well‐known model of tumorigenesis in chronic myeloproliferative diseases and certain leukemias 9, but the role of STAT5 in lymphocyte development and transformation is not so well understood.

The implication of STAT3 and STAT5 in leukemias and lymphomas that are correlated with viruses has been long speculated, and there are several in vitro studies in the literature that prove this point. As an example, human T‐cell lymphoptropic virus (HTLV)‐related leukemogenesis has been correlated with the induction of STAT1, 3, and 5 by the virus, and the JAK‐STAT pathway has been found to be constitutively activated in T cells transformed with HTLV‐1 10, 11. Moreover, it has been shown that STAT3 and STAT5 are induced by Tax (transactivator from the X‐gene region) 12, 13, the main oncoprotein of HTLV‐1 and 2. Similar observations have been made in HIV‐infected T cells 14, 15, 16.

Epstein–Barr Virus (EBV)‐related lymphomagenesis has not been thoroughly studied in correlation with the JAK‐STAT pathway, but there are in vitro data that support a linkage between the presence of the virus and STAT expression in tumors. In 1996, researchers noted that there is a constitutive activation of STAT proteins in EBV‐positive lymphoblastoid and Burkitt lymphoma cell lines, and that activation of the lymphoblastoid EBV‐positive cells coincided with cellular interleukin (IL) ‐10 expression and high Bcl‐2 levels 17. Constitutive activation of the JAK‐STAT pathway has also been demonstrated in spontaneous lymphoblastoid cell lines derived from patients with EBV‐related posttransplant lymphoproliferative disease 18, and was found to be provoked by the virus autocrine interleukin‐10 production, a process that is essential for the proliferation of the infected cells and the lymphomagenesis cascade 19.

The STAT family has been studied in chronic lymphocytic leukemia (CLL) and several investigators have shown that STAT proteins are phosphorylated—thus activated, in cells derived from patients with CLL 20, 21. Moreover, it has been shown that STAT3 activates NF‐kB in CLL cells, which seems to play a major role in the pathogenesis of the disease 22, and that it can regulate microRNA gene expression in CLL cells 23. The JAK‐STAT3 pathway has been experimentally activated in CLL cells by stimulation of the B‐cell receptor, while treatment of these cells with the JAK1/2 inhibitor ruxolitinib has been found to inhibit STAT3 phosphorylation and to induce apoptosis of the cells 24. Other JAK inhibitors have been shown to be effective in inhibiting the STAT1 pathway and to strongly induce apoptosis of CLL cells 25.

Activation of the IL‐10 receptor expressed on CLL cells has been shown to result in the phosphorylation of STAT1 and STAT3 26. Since EBV‐enhanced IL‐10 production is a major step in the interactions of EBV with the B and T cells and constitutes a central event in the pathogenesis of B‐cell transformation 19, IL‐10 upregulation by the virus could be related to STAT expression in EBV‐positive patients with CLL. The literature lacks studies of STAT5 expression in CLL, and recent studies 27, 28, 29, have shown a possible participation of the virus in the pathogenesis and the clinical course of CLL. In light of these facts, a correlation of STAT5 expression with EBV would enrich our knowledge on the role of the virus in the pathogenesis of CLL. We chose to study the expression of total STAT5 and STAT5b because these proteins have never been studied in CLL. Moreover, most of the studies on STAT proteins are based on the detection of STAT phosphorylation and not expression. We decided to study the expression of STAT5/5b based on the speculation that STAT activation correlates well with the upregulation of STAT‐encoding genes, and thus, the expression of STAT proteins.

We investigated the possible correlations of the presence of EBV and its major oncoprotein (LMP1) with the expression of STAT5 and STAT5b in patients with CLL. Correlations with several prognostic factors were also carried out, in order to identify a possible prognostic role of the levels of STAT5/5b in CLL and the impact of the detection of the virus on these parameters.

## Methods

Informed consent was obtained from 63 patients with CLL and 15 healthy blood donors. All patients had immunophenotypically confirmed disease by peripheral blood at the time of sample collection.

Peripheral whole blood samples were collected in ethylenediaminetetraacetic acid. Within 6 h from collection, DNA extraction was performed with the QIAamp^®^ DNA Blood mini kit (Qiagen, Venlo, the Netherlands). The Trizol protocol (Invitrogen, Carlsbad, CA) was used to extract and purify total RNA. Reverse transcription was performed using MMLV‐derived reverse transcriptase enzyme (Invitrogen), according to standard protocols. For protein extraction, radioimmunoprecipitation assay buffer was added to the extracted white blood cells and lysates were incubated at 0°C, centrifuged and eventually stored at −80°C.

### EBV DNA Quantification

Quantification of the viral load was based on the detection of the *BXLF1* thymidine kinase gene of the virus, using the EBV R‐gene^™^ Quantification COMPLETE kit (bioMérieux, Paris, France) on LightCycler^®^ 2.0 (ROCHE, Mannheim, Germany) according to the manufacturer's instructions. The detection limit of the reaction is <4 copies/PCR, and the results are expressed in viral copies/mL of sample.

### Conventional RT‐PCR for the detection of LMP1‐mRNA

Primers and probes were chosen for *LMP1* 30 and the stably expressed housekeeping Abelson murine leukemia viral oncogene (*ABL1*) 31 and the reactions were carried out in a gradient cycler (Mastercycler Gradient, Eppendorf, Hamburg, Germany) using the conditions provided by previous authors 30. A novel human cell line (MDA‐V), established from a patient with EBV‐positive classical nodular sclerosing Hodgkin lymphoma 32, and Karpas 299, an anaplastic large‐cell lymphoma cell line were used as positive and negative controls, respectively. The LMP1 PCR product of 102 bp was verified with direct sequencing. LMP1 expression in the sera of all PCR‐LMP1‐positive patients was verified with the use of ELISA (LMP1 detection kit, MYBiosource, San Diego, CA), according to a standard protocol.

### Cell line source, testing, and characterization

Both cell lines were a kind offer of Professor G. Rassidakis, MD, PhD, University of Athens. They were obtained just before the initiation of the PCR experiments, in May 2013, and they were last tested just before obtaining them.

The MDA‐V cell line was established from a patient with Ann Arbor stage‐I classical Hodgkin lymphoma, diagnosed and treated at the University of Texas M.D. Anderson Cancer Center (Rassidakis et al., manuscript in preparation). The diagnosis of classical Hodgkin lymphoma, nodular sclerosis type, was made on a lymph node of the patient according to the WHO classification criteria. The neoplastic Hodgkin cells were positive for the EBER1/2 transcripts of the EBV virus in the initial (diagnostic) lymph node tissue. Suspension cells from fresh lymph node tissue, obtained at the time of diagnosis prior to treatment, were immediately put in culture for 2 days and then the cells were injected subcutaneously in SCID mice. Tumor cells from the xenografts were subcultured and were injected again in SCID mice for four times before the cell line was established. The MDA‐V cells were tested positive for EBV at the DNA and RNA level by real‐time polymerase chain reaction (RT‐PCR) and PCR methods, respectively. The immunophenotype of the cells was identical to that of the diagnostic lymph node as assessed by immunohistochemistry on the MDA‐V cell blocks. The MDA‐V cell line carries wild‐type p53 gene 32 and is characterized by increased AP‐1 activity with high expression of JNK and cJUN proteins as well as NF‐Kb activation. Whole genome sequencing is ongoing to assess the complete mutation status of the MDA‐V cell line.

Karpas 299 (K299) cell line was characterized in 1988 33. The line was established from blast cells in the peripheral blood of a 25‐year‐old white man. His illness, which began with enlarged occipital and axillary nodes and weight loss, ended after 7 months with generalized lymphadenopathy, pleural effusion, and bone marrow involvement. A lymph node biopsy showed a large‐cell lymphoma mainly sinusoidal in distribution. The blast cells with pleomorphic nuclei resembled primitive histiocytes. The cells, which expressed the T‐cell‐associated markers CD4 and CD5, were positive for HLA‐DR, epithelial membrane antigen, and CD30 (Ki‐1 antigen). The karyotype was aneuploid and included a translocation 2;5. The site of translocation on chromosome 5 (at 5q35.1) is in the region of the locus of the c‐fms oncogene (receptor of the monocyte‐macrophage colony‐stimulating factor MCSF or CSF‐1). The cell line Karpas 299 has the same karyotype and pattern of antigen expression as the patient's cells. Northern blot analysis of RNA showed an active rearrangement of the T‐cell receptor beta‐chain gene.

### Western blotting

Protein extracts were separated on 10% w/v SDS‐PAGE; the PVDF membranes were blocked in 5% milk TPBS 0.1% and incubated with anti‐STAT5b antibody [(G‐2) sc‐1656 mouse monoclonal IgG] (1:800) overnight. Following TPBS 0.1% washing, anti‐mouse antibody (1:10000) was applied and after another washing, the immunoreactive bands were detected by enhanced chemiluminescence with ECL (Cell Signaling) according to the manufacturer's instructions, using luminescent image analyzer (Fujifilm LAS‐4000). The procedure was repeated after stripping, and reprobing each PVDF membrane using anti‐STAT5 [(C‐17) sc‐835 rabbit polyclonal IgG] (1:800) and anti‐rabbit (1:10000) antibodies. Anti‐actin antibody was used to control protein loading and rat whole brain extracts were used as controls for STAT5/STAT5b expression. The secondary antibodies used were affinity‐purified antibody, peroxidase‐labeled goat anti‐rabbit and anti‐mouse IgG (H‐L) (Kirkegaard & Perry Laboratories, Gaithersburg, Maryland USA). Gel Pro Analyzer 4 was used to analyze the results.

### Statistical analysis

IBM SPSS statistics, v19.0 (IBM Corporation, Armonk, North Castle, NY) was used, and the individual methods are provided with each result.

## Results

Sixty‐three patients with CLL with immunophenotypically confirmed disease by peripheral blood at the time of sample collection were included in the study. Their demographic and basic clinical and laboratory characteristics are shown in Table 1. The vast majority of the patients (51/63, 81%) were treatment naïve and according to the Binet staging system for CLL 34, 27 (42.9%) had clinical stage A disease, 22 (34.9%) stage B, and 14 (22.2%) stage C.

**Table 1:** Patients’ demographic, clinical, and hematologic characteristics

Characteristics	Result
Number of patients, *N* (%)	63 (100)
Sex (Male to female ratio)	1.52
Age (years), median (range)	69 (37–91)
Clinical stage (Binet), *N* (%)	
A	27 (42.9)
B	22 (34.9)
C	14 (22.2)
Previous treatment, *N* (%)	12 (19.0)
Peripheral blood lymphocytes (×10^9^/L), median (range)	14.1 (1.4–131)
LDH/ULN, Median (range)	1.16 (1–8)
Hemoglobin (g/dL), Median (range)	13.3 (8.3–16.1)
Platelets (×10^9^/L), median (range)	168 (30–326)
B_2_‐microglobulin/ULN, Median (range)	2.7 (0.2–11.9)

LDH, lactic dehydrogenase; ULN, upper limit of normal.

Twenty‐two (34.9%) patients tested positive by PCR for EBV and the median detected viral load was 2640.7 copies/mL (range 580.0–4248.1). LMP1‐mRNA was detected by PCR in 14/22 (63.6%) patients, and the LMP1 oncoprotein was detected by ELISA in all PCR‐positive samples. No difference in EBV positivity was noted between treatment naïve (7/51, 13.7%) and previously treated patients (5/12, 41.6%) (*P* = 0.062).

**Figure 1:**
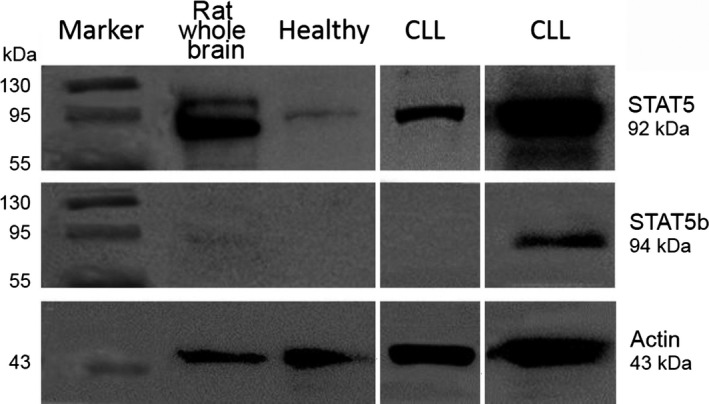
Protein immunoblot results. The figure includes the immunoblot results of three subjects. One healthy blood donor expressing STAT5 but not STAT5b (none of the healthy subjects expressed STAT5b), one patient with CLL expressing STAT5 but not STAT5b, and one patient with CLL expressing both total STAT5 and STAT5b. STAT, signal transducer and activator of transcription; CCL, chronic lymphocytic leukemia.

Total STAT5 expression (expressed as a STAT5 to actin ratio) was detected in 38 patients (60.3%) and STAT5b expression (expressed as a STAT5b to actin ratio) was detected in 18 patients (28.6%). The corresponding values in healthy controls were 8 (57.1%) and 0 (0%) and the statistical significance of the differences between patients and healthy controls was 0.827 and 0.022 (Pearson's chi‐square, two‐sided *P*). There was a nonstatistically significant trend for higher total STAT5 expression in patients than in healthy individuals. These results are shown in detail in Table 2. Figure 1 shows some indicative results of the protein immunoblot for total STAT5 and STAT5b in healthy subjects and patients with CLL.

**Table 2:** STAT5 and STAT5b expression in patients and healthy controls

	Patients	Healthy controls	*P*
STAT5 expression, *N* (%)	38 (60.3)	8 (57.1)	0.827^a^
Level of STAT5 expression, Median (range)	1.19 (0.24–2.38)	0.52 (0.29–0.96)	0.095^b^
STAT5b expression, *N* (%)	18 (28.6)	0 (0)	0.022^a^
Level of STAT5b expression, Median (range)	0.22 (0.01–1.80)	0 (0)	NA

a Pearson chi‐square, two‐sided *P*.

b Independent Samples Mann–Whitney *U*‐test, two‐sided *P*.

The expression of STAT5 and STAT5b was tested against the age and sex of the patients, stage of the disease, peripheral blood lymphocyte count, hemoglobin concentration, platelet count, levels of lactic dehydrogenase (LDH) and b2‐microglobulin, and previous treatment status. The results are recorded in Table 3. The only statistically significant correlations noticed were that of STAT5 expression with the concentration of hemoglobin −12.5 g/dL in STAT5‐positive versus 13.9 g/dL in STAT5‐negative patients (independent samples Mann–Whitney *U*‐test, two‐sided *P* = 0.002), and those of STAT5 and STAT5b expression with the Binet clinical stage (Pearson's chi‐square test, two‐sided *P* = 0.009 and 0.002, respectively).

**Table 3:** Correlation of STAT5 and STAT5b expression with the basic clinical and laboratory characteristics of the patients

Value	All patients	STAT5(+)	STAT5(−)	*P*	STAT5b(+)	STAT5b(−)	*P*
Number of patients, *N* (%)	63 (100%)	38 (60.3)	25 (39.7)	NA	18 (28.6)	45 (71.4)	NA
Age, Mean (range)	68.9 (37–91)	70.1 (37–91)	67.1 (54–86)	0.19^b^	70.2 (37 – 87)	68.4 (49–91)	0.24^b^
Male to female ratio	1.52	1.37	1.78	0.63^c^	1.57	1.5	0.94^c^
Stage (Binet), *N* (%)							
A	27 (44.3)	11 (28.9)	17 (68.0)	0.009^c^	2 (11.1)	26 (57.8)	0.002^c^
B	20 (32.8)	16 (42.1)	5 (20.0)		8 (44.4)	13 (28.9)	
C	14 (23.0)	11 (28.9)	3 (12.0)		8 (44.4)	6 (13.3)	
Hemoglobin concentration (g/dL), Mean (range)	13.1 (8.3–16.1)	12.5 (8.3–15.5)	13.9 (9.7–16.1)	0.002^b^	12.5 (10.0–15.5)	13.3 (8.3–16.1)	0.09^b^
Peripheral blood lymphocytes (×10^9^/L), Mean (range)	25.1 (1.4–131.0)	25.2 (1.4–110.1)	25.0 (1.85–131.0)	0.54^b^	23.2 (1.4–79.6)	25.9 (1.9–131.0)	0.96^b^
Platelet count (×10^9^/L), Mean (range)	168.9 (30–326)	160.1 (30–326)	180.6 (52–257)	0.19^b^	149.7 (30–246)	176.8 (52–326)	0.21^b^
LDH/ULN, Mean (range)	1.52 (1–8)	1.74 (1–8)	1.25 (1–3)	0.21^b^	2.08 (1–8)	1.3 (1–3)	0.36^b^
b2‐microglobulin/ULN, Mean (range)^a^	3.3 (0.2–11.9)	3.8 (0.2–11.9)	2.5 (1.6–4.8)	0.13^b^	4.1 (0.2–11.9)	2.9 (1.6–6.17)	0.42^b^
Previous treatment, *N* (%)	51 (80.9)	7 (58.3)	5 (41.7)	0.56^c^	5 (41.7)	7 (58.3)	0.22^c^

NA, not applicable; LDH, lactic dehydrogenase; ULN, upper limit of normal; STAT, signal transducer and activator of transcription.

a Available in 33 patients.

b Independent samples Mann–Whitney *U*‐test, two‐sided *P*.

c Pearson's chi‐square test, two‐sided *P*.

Moreover, there was a statistically significant correlation of STAT5 and STAT5b expression to the presence of EBV and to LMP1 expression, as shown in Table 4. In detail, EBV‐positive (by PCR) patients expressed STAT5 in a significantly higher proportion than EBV‐negative patients (77.3% vs. 51.2%, Pearson's chi‐square test, two‐sided *P* = 0.044), and the same but even more pronounced result was found for STAT5b (50.0% vs. 17.1%, Pearson's chi‐square test, two‐sided *P* = 0.006). LMP1‐positive (by PCR and Western blot analysis) patients expressed STAT5 in a higher percentage than LMP1‐negative patients (91.7% vs. 52.9%, Pearson's chi‐square test, two‐sided *P* = 0.014), and again the same applied for STAT5b (58.3% vs. 21.6%, Pearson's chi‐square test, two‐sided *P* = 0.011).

**Table 4:** Correlation of EBV and LMP1 status with the expression of STAT5 and STAT5b

Patients	EBV(+), *N* (%)	EBV(−), *N* (%)	*P* a
Expressing STAT5	17 (77.3)	21 (51.2)	0.044
Not expressing STAT5	5 (22.7)	20 (48.8)	
Expressing STAT5b	11 (50.0)	7 (17.1)	0.006
Not expressing STAT5b	11 (50.0)	34 (82.9)	
	LMP1(+)	LMP1 (−)	
Expressing STAT5	11 (91.7)	27 (52.9)	0.014
Not expressing STAT5	1 (8.3)	24 (47.1)	
Expressing STAT5b	7 (58.3)	11 (21.6)	0.011
Not expressing STAT5b	5 (41.7)	40 (78.4)	

EBV, Epstein‐Barr virus; LMP1, latent membrane protein 1.

a Pearson's chi‐square test, two‐sided *P*.

**Figure 2:**
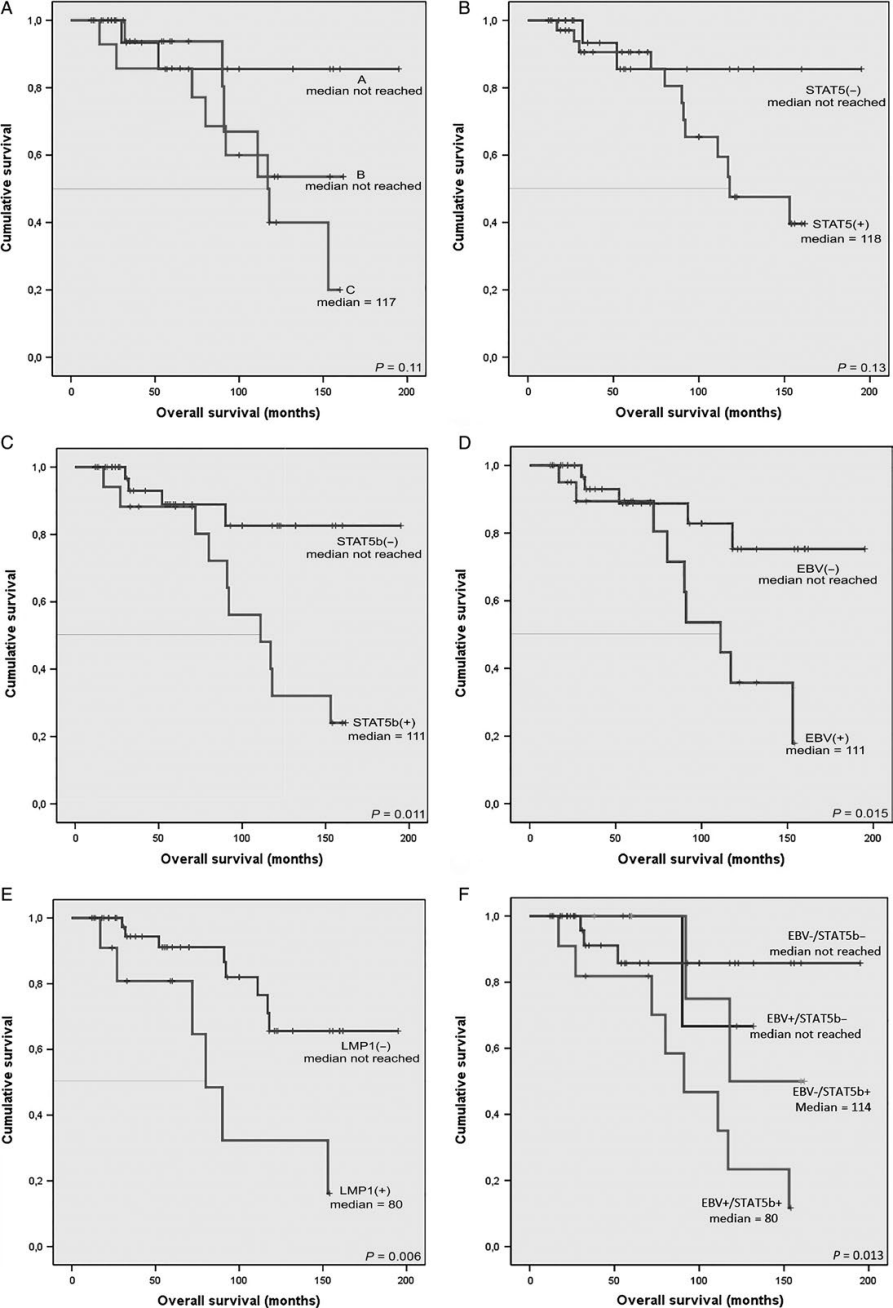
Kaplan–Meier survival curves. (A) Survival by Stage (Binet Clinical Staging) 33; (B) EBV‐positive versus EBV‐negative patients; (C) LMP1‐positive versus LMP1‐negative patients; (D) STAT5‐positive versus STAT5‐negative patients; (E) STAT5b‐positive versus STAT5b‐negative patients; (F) Survival of patients based on the combined EBV and STAT5b status. EBV, Epstein–Barr virus; STAT; signal transducer and activator of transcription.

The patients were prospectively followed according to common clinical practice for a median period of 27 (5–68) months since sample collection. Five (7.9%) patients were lost to follow‐up, and 14 deaths were recorded during the follow‐up period. Some significant survival correlations were noted. The correlation of overall survival (OS) to the clinical stage was not statistically significant, as shown in the corresponding Kaplan–Meier curve (Fig. 2A). The median survival for each stage was as follows: stage A, not reached, stage B, not reached, and stage C, 117 months (log‐rank test, *P* = 0.11). On the other hand, OS was strongly correlated with the detection of the virus and the expression of LMP1 (Fig. 2B and C). The median OS of EBV‐positive patients was 111 months, while that of EBV‐negative patients was not reached (log‐rank test, *P* = 0.015). An even stronger correlation was found between OS and LMP1 expression. LMP1‐positive patients had a median OS of 80 months, while the median OS of LMP1‐negative patients was not reached (log‐rank test, *P* = 0.006). The median OS of patients expressing STAT5 (118 months) did not differ in a statistically significant way from that (not reached) of patients not expressing STAT5 (log‐rank *P* = 0.13), but patients expressing STAT5b had a significantly lower (111 months) OS than patients not expressing STAT5b (not reached) (log‐rank test, *P* = 0.011). The corresponding curves are shown in Figure 2(D and E).

We also studied the OS of four different groups of patients, based on the presence of the virus and the expression of STAT5b (11 EBV+/STAT5b+, 11 EBV+/STAT5b‐, 7 EBV‐/STAT5b+, and 34 EBV‐/STAT5b‐ patients). As shown in Figure 2F, OS of EBV+/STAT5b+ patients was the lowest, while OS of EBV‐/STAT5b‐ patients was the highest (log‐rank test, *P* = 0.013).

## Discussion

The WHO classification of Tumours of Haematopoietic and Lymphoid Tissues of 2008 35 has recognized several EBV‐related B‐cell lymphoproliferative disorders, characterized by LMP1 and EBV nuclear antigen (EBNA) positivity, as part of latency type 3 of the virus. This type of latency characterizes neoplasms arising in the context of decreased host immunosurveillance. CLL does not belong to the EBV‐related lymphoproliferative disorders, but there are several studies implying that EBV may play a role in the pathogenesis, course and prognosis of the disease.

Although preliminary reports had proposed that CLL B cells are rather resistant to the transforming actions of the virus 36, LMP1 has been previously detected in B cells from patients with CLL 28, 29, 37, but the role of the virus in the pathogenesis of the disease has not yet been clarified, although its presence has been related to a more aggressive course and to Richter's transformation 38, 39, 40. Nevertheless, none of the studies conducted to date has addressed the topic of viral lymphomagenesis in CLL, and there are no data about the role of the virus in the prognosis of the disease.

On the other hand, there are several studies correlating STAT proteins to the presence of EBV in various neoplasms. STAT1 and STAT3 have been found to be constitutively activated in EBV‐positive lymphoblastoid and Burkitt's lymphoma cell lines 17. LMP1, the major oncoprotein of EBV, acts as a constitutively active functional mimic of CD40 41. It has been shown that LMP1 through its C‐terminal activating region can activate JAK3 leading to activation of the STAT proteins 42, and that EBV‐infected type III latency cells, where LMP1 is a major component of the latency, express high levels of STATs (STAT1, 2, 3, 5A) 43. It has been also shown that there is a linkage between STAT regulation and EBV gene expression in tumors. The constitutively active STAT proteins can regulate LMP1 expression through two LMP1 promoters (L1‐TR 44 and BamHI‐Q 45), while LMP1 has been shown to activate STATs. These two interactions seem to be concomitantly active, forming a positive autoregulatory loop of LMP1 expression and STAT activation that has been documented in epithelial cells infected by EBV 46.

These interesting interactions seem to be confirmed by our results in CLL, where EBV and LMP1 expression are correlated with the expression of STAT5 and STAT5b. STAT5 was overexpressed in patients in comparison to healthy controls, but STAT5b was exclusively expressed in patients, since none of the healthy blood donors was STAT5b‐positive. This sharp expression of STAT5b in CLL cells but not in normal lymphocytes should be further confirmed and evaluated in larger patient series, in order to provide more information about the potential role of STAT5b in the pathogenesis and course of the disease.

According to our results, EBV and LMP1‐positive patients had a significant survival disadvantage in comparison to EBV and LMP1‐negative patients. The same survival disadvantage was found for STAT5b‐positive in comparison to STAT5b‐negative patients. Moreover, double positivity (for EBV and STAT5b) was correlated with a worse prognosis, than positivity for one of the two parameters, while double negative patients had the highest overall survival from all four patient groups, and this result was of statistical significance (*P* = 0.013). These strong correlations pose a new perspective in the research of CLL pathogenesis, and this is the first, to the best of our knowledge, report of a survival disadvantage of EBV‐positive patients with CLL. None of the studied EBV‐positive patients progressed to Richter's transformation, during the follow‐up period.

Moreover, STAT5 and STAT5b seem to be expressed in patients with more advanced disease, a fact that renders their expression a strong prognostic factor in CLL. This result should be further studied in order to reach a more rigid conclusion. It is noteworthy though, that STAT5b expression was strongly correlated with OS in our cohort of patients, and this correlation was stronger than that of the Binet clinical staging of CLL to OS. This result should not be overlooked for one more reason. STATs are potential treatment targets, and their expression in CLL could define a subcategory of patients that could benefit from such a targeted treatment.

In conclusion, our results provide one more clue about the implication of EBV in the pathogenesis and course of CLL, a fact that becomes more and more evident with time. The correlation of the presence of the virus and its major oncoprotein with the expression of STAT5 proteins and especially STAT5b, and the lower survival of EBV and LMP1 positive patients probably highlights another interesting case of viral lymphomagenesis.

## Conflict of Interest

No conflict of interest to disclose.
